# Referral Patterns of General Dental Practitioners for Implant Surgery Procedures

**DOI:** 10.3290/j.ohpd.b4438887

**Published:** 2023-09-27

**Authors:** Adrian Kahn, Daya Masri, Lazar Kats, Roni Kolerman, Sarit Naishlos, Tom Shmuly, Dror Allon, Liat Chaushu

**Affiliations:** a Oral and Maxillofacial Surgeon, Department of Oral and Maxillofacial Surgery, Goldschleger School of Dental Medicine, Tel-Aviv University, Ramat Aviv, Israel; Department of Oral and Maxillofacial Surgery, Rabin Medical Center, Petach-Tikva, Israel. Conceptualisation, methodology, investigation, wrote the manuscript, reviewed and edited manuscript, supervision. Read and agreed to the published version of the manuscript.; b Oral and Maxillofacial Surgeon, Department of Oral and Maxillofacial Surgery, Rabin Medical Center, Petach-Tikva, Israel. Conceptualisation, visualisation, investigation, resources, wrote the manuscript, reviewed and edited manuscript. Read and agreed to the published version of the manuscript.; c Specialist in Oral Medicine, Department of Oral Pathology, Oral Medicine and Maxillofacial Imaging, Goldschleger School of Dental Medicine, Tel-Aviv University, Ramat Aviv, Israel. Investigation, resources, data curation. Read and agreed to the published version of the manuscript.; d Periodontist, Department of Periodontology and Implantology, Goldschleger School of Dental Medicine, Tel-Aviv University, Ramat Aviv, Israel. visualisation, validation, wrote the manuscript, reviewed and edited manuscript, supervision. Read and agreed to the published version of the manuscript.; e Pedodontist, Department of Pediatric Dentistry, Goldschleger School of Dental Medicine, Tel-Aviv University, Ramat Aviv, Israel. Software, validation, formal analysis, investigation, resources, data curation, reviewed and edited manuscript. Read and agreed to the published version of the manuscript.; f Oral and Maxillofacial Surgeon, Department of Oral and Maxillofacial Surgery, Goldschleger School of Dental Medicine, Tel-Aviv University, Ramat Aviv, Israel; Department of Oral and Maxillofacial Surgery, Rabin Medical Center, Petach-Tikva, Israel; Department of Oral and Maxillofacial Surgery, Kaplan Medical Center, Rehovot, Israel. Visualisation, formal analysis, resources, data curation. Read and agreed to the published version of the manuscript.; g Oral and Maxillofacial Surgeon, Department of Oral and Maxillofacial Surgery, Goldschleger School of Dental Medicine, Tel-Aviv University, Ramat Aviv, Israel. Validation, visualisation, investigation, resources, data curation. Read and agreed to the published version of the manuscript.; h Periodontist, Department of Periodontology and Implantology, Goldschleger School of Dental Medicine, Tel-Aviv University, Ramat Aviv, Israel. Conceptualisation, visualisation, methodology, software, validation, formal analysis, data curation, wrote the manuscript, reviewed and edited manuscript, supervision. Read and agreed to the published version of the manuscript.

**Keywords:** dental implant, implant placement, referral patterns

## Abstract

**Purpose:**

The growing demand for implants has led to their implementation by general dental practitioners (GDPs) in clinical practice. The present study assessed referral patterns of GDPs for the surgical phase of implant dental treatment.

**Materials and Methods:**

One hundred fifty GDPs were asked to fill out a structured questionnaire containing their demographic data and answer six questions characterising their referral patterns for implant dentistry.

**Results:**

Forty-one (41%) percent performed the surgical phase, and 87% provided implant restoration. Gender was the only influencing factor for the surgical phase, as 51.4% of male GDPs and 6.5% of female GDPs performed implant surgery themselves. Experience and practice set-up did not influence the referring decision. Fifty-four percent of the practitioners referred 0 to 5 patients per month, and the chosen specialists were: 80% oral and maxillofacial surgeon, 11% periodontist, and 9% selected a specialist depending on the individual case. The major reasons influencing the referral pattern were the complexity of the surgical procedure, followed by systemic medical compromise of the patient.

**Conclusions:**

Most implant surgeries in Israel are still performed by specialists.

In medicine and dentistry, general practitioners (GP) and general dental practitioners (GDP) provide primary care. In medicine, 85% of physicians are specialists.^22^ The abundance of medical specialists has made it easy for individuals to self-refer to medical specialists. In dentistry, however, only 20% of dentists are certified specialists.^1,2^ Moreover, it is more difficult for a patient to approach the exact dental specialist who will provide proper treatment. Consequently, it is uncommon for dental patients to self-refer to most dental specialists.

As a result, patients who require oral care generally seek help and advice from a GDP, who will make the decision whether s/he will provide the dental care by her/himself or will further refer the patient to be treated by a specialist. For the past three decades, implant dentistry has been an evolving field in oral care. However, there is still a lack of consensus regarding who should perform implant surgery.

In the 1970s and 1980s, Branemark’s group approach allowed only surgical specialists to participate in courses training for implant surgery. Therefore, implant surgery was initially the exclusive domain of oral and maxillofacial surgeons until 1990. Since then, periodontists have also begun to perform implant therapy. During the past 10 years, there has been a clear trend for other dental practitioners (e.g., general dentists, prosthodontists, and endodontists) to become increasingly more involved in implant surgery. This trend is also encouraged by the implant industry.^3,9,11^

Implant dentistry has become the preferred treatment alternative in cases of missing teeth.^3-6^ Preoperative expectations are extremely important when assessing how patients perceive the outcomes. Patient-reported oral health-related quality of life outcomes are crucial. In the majority of cases, patients’ reports are incongruent with clinicians’ evaluations.^10^

Implant dentistry aims to restore function and aesthetics in the long term.^3^ Implant failure may affect the treatment plan and its longevity,^4-6^ and may be early or late. The aetiology and risk factors of implant failure are numerous.^20^ Age, comorbidities, oral hygiene, and restoration quality may influence treatment outcome. Adverse outcomes can be avoided by evaluating medical history, medications, functional needs, and designing restorations that allow access for hygiene. Implant risk-assessment tools show promise by providing a systematic approach for early diagnosis to avoid future complications.^14,17-19^

Peri-implantitis, its risks and protective indicators are still a research challenge.^4-6,21^ The prevalence in a university cohort was 10.7% at the implant level and 21.3% at the patient level.^21^

The human factor and its relation to early implant failure is often ignored. Poor technique or wound healing are mentioned as principal factors. Surprisingly, even the best practitioners may fail. It is not the skills but rather the application of the knowledge that leads to the final result. Organisational factors as checklists might reduce early failures.^19^

Health organisations such as national health care services or national dental associations, are significant providers of medical dental care. Their increasing involvement in dentistry must take the GDP into consideration as the architect of the dental treatment from which patient care starts. The purpose of the present survey was to assess referral patterns of GDPs for the surgical phase of implant dentistry to allow future resource planning regarding implant dentistry.

## Materials and Methods

This study was conducted according to the guidelines of the Declaration of Helsinki and approved by the Ethics Committee of Tel Aviv University.

At the Israeli national annual dental congress, an independent research stand was used, at which dentists were asked to fill out a questionnaire for research purposes. Out of the 2000 dentists who attended the congress, 150 GDPs agreed to participate in the survey. At the meeting, they received a cover letter stating the objectives of the study and a questionnaire (Appendix A) with items covering their demographic data and six questions characterising their referral patterns for the surgical phase of implant dentistry. The questions addressed were: GDP performance of implant surgery, GDP performance of implant rehabilitation, the frequency of patient referral, the parameters influencing the decision of the GDP for referral, the specialist to whom the patient will be referred, and the type of clinical institution preferred. GDPs could answer whether they would treat the patients themselves or refer them to a specialist. GDPs who chose referral in their answer were further asked to select the specialist group to which they would refer the patient and specify reasons for referral. The study was approved by the Tel Aviv University ethics committee.

### Statistical Analysis

An independent statistician performed data analysis using SPSS software (IBM; Armonk, NY, USA). Statistical analysis included the Χ^2^ test and Fisher’s exact test. These tests were used to determine whether an association existed between nominal and categorical data in the examined population. The present study tested only a few categorised groups (e.g., female vs male; referring vs non-referring dentists). Each group was designated by a number (e.g., female = 0, male = 1). All the examined parameters were analysed according to the questionnaire, in which every answer was given a specific number that was later analysed by the statistical test.

## Results

One hundred forty (140) out of 150 GDPs returned the completed questionnaire for a response rate of 93%. Seventy-eight percent (109/140) of the responders were males, and 22% (31/140) were females. The mean age of respondents was 47 ± 12 years (range 27–79 years).

Forty-one percent (41%) of the GDPs performed the surgical phase of implant therapy themselves (Table 1). Ninety-three percent (93%) of the responding practitioners provided the restorative phase (Table 2). Six and a half percent (6.5%) of the females vs 51% of the males performed surgery (p < 0.001) (Table 3).

**Table 1:** Rate of implant surgery performed

Surgical phase	No	Yes	Total
Total	82	58	140
	59%	41%	100%

**Table 2:** Rate of of implant prosthetics performed

Prosthetic phase	No	Yes	Total
Total	18	122	140
	6.8%	93.2%	100%

**Table 3:** Rate of implant surgery performed according to gender

Gender	No	Yes	Total
Females	29	2	31
	93.5%	6.5%	100%
Males	53	56	109
	48.6%	51.4%	100%

No significant differences regarding the restorative phase were noted between females (81%) and males (92%) (p = 0.062) (Table 4). Gender was an influencing factor merely for the surgical phase.

**Table 4:** Rate of implant prosthetics performed according to gender

Gender	No	Yes	Total
Females	6	25	31
	19.35%	80.64%	100%
Males	9	100	109
	8.3%	91.7%	100%

Years of professional experience was not a statistically significant influencing factor. The mean was 17 years for those GDPs performing surgery vs 20 years for those who did not provide surgical implant therapy (p = 0.083).

**Fig 1:**
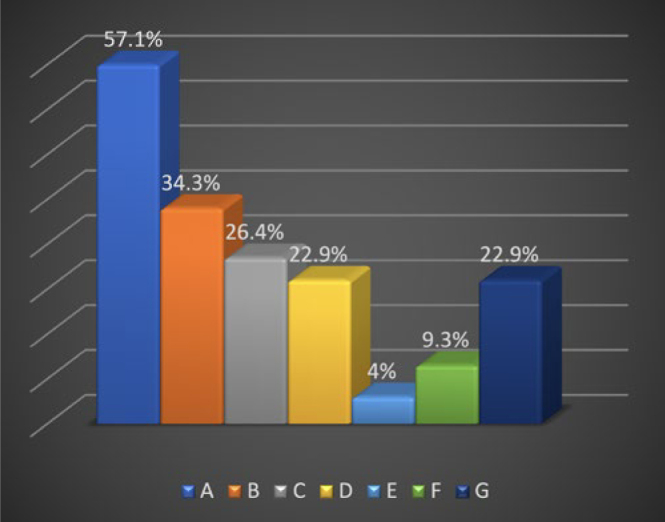
Reasons for referral to implant therapy. A: The complexity of the surgical procedure; B: the systemic medical status of the patient; C: the specialist’s expertise; D: the GDP does not perform implant surgery on a routine basis; E: inadequate available surgical instruments; F: collaboration with a specialist clinic; G: concern about post-operative complications.

Reasons for patient referral for implant surgery included the complexity of the surgical procedure 57%, medically compromised patients 34%, and the enhanced expertise of the specialist 26%. Every responder could choose more than one option (Fig 1).

**Fig 2:**
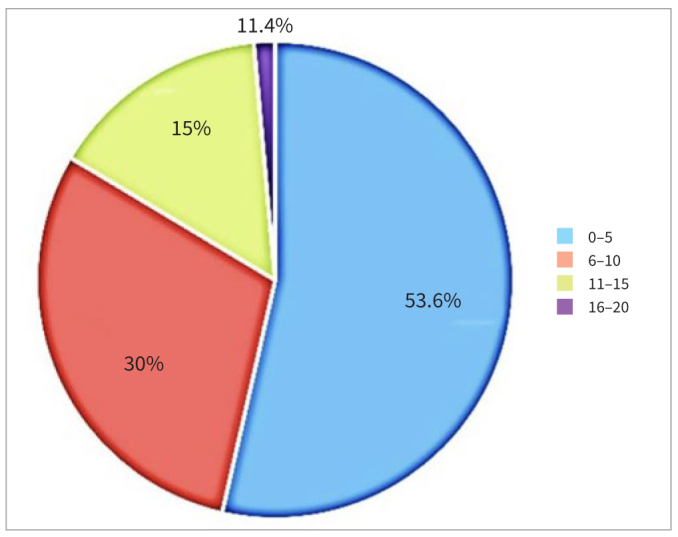
Number of surgery referrals per month.

Fifty-four percent (54%) of the practitioners referred 0–5 patients per month, 30% referred 6–10, 15% referred 11–15 and 1.4% referred 16–20 patients every month (Fig 2).

**Fig 3:**
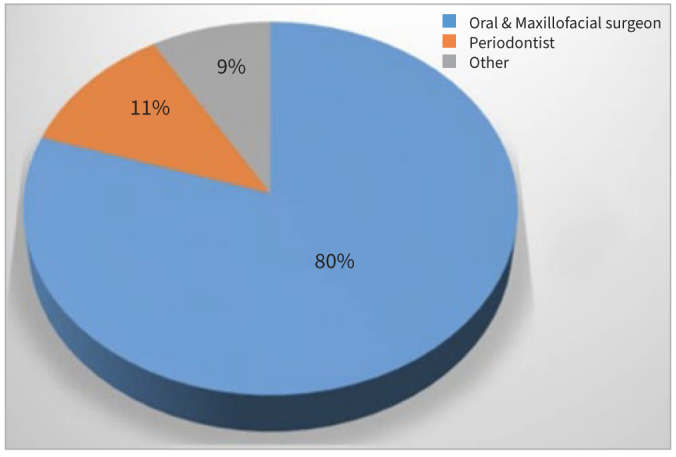
Proportion of specialists chosen for implant placement.

In terms of the specialist chosen by the GDP for implant surgery, 80% chose oral and maxillofacial surgeons, 11% chose periodontists, and 9% chose the specialist according to the individual case (Fig 3).

**Fig 4:**
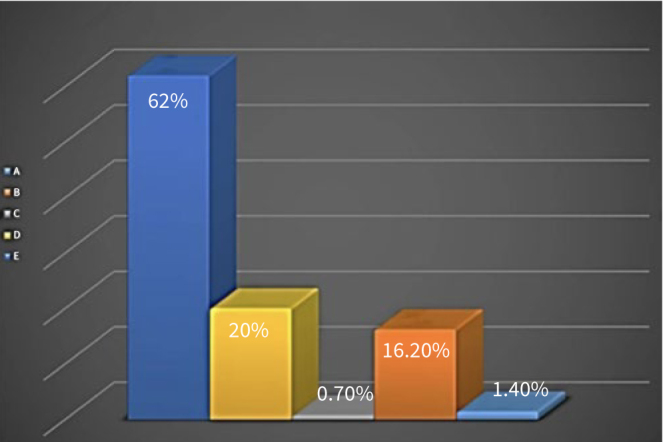
Characteristics of referral clinics. A: the clinic invites a specialist for specific cases; B: Hospital, Oral, and Maxillofacial Surgery Department; C: University, Oral and Maxillofacial Surgery Department; D: specialist’s clinic; E: doesn’t matter to which clinic as long as the treatment is performed by a specialist.

Regarding the referral clinic, 62% of the responding GDPs had no preference about clinic characteristics as long as the patient is treated by a specialist, 20% opted for a specialist working in a private clinic, and 16% preferred the oral and maxillofacial department in a hospital (Fig 4).

The data supporting this study’s findings are available from the corresponding author [AK] upon reasonable request.

## Discussion

This survey study assessed referral patterns and factors that inﬂuence a GPD’s decision to refer patients for implant surgery. According to the present survey, at least 40% of GDPs performed implant surgery themselves. This is substantially higher than the only 16% of Virginia (USA) GDPs who performed the surgical phase of implant therapy in a 2007 survey,^7^ or merely 10% in a study performed by Boston University dental school graduates.^8^ We can clearly see that the yearly difference between the surveys demonstrates that more and more GDPs are engaging in the surgical part of implant dentistry. This might lead to allowing GDPs to perform implant surgery for healthcare organisations (e.g., national healthcare services), making implant dentistry more available to patients.^11,12^

According to our questionnaire, nearly 90% of GDPs perform implant restorations. This is in contrast with only 61% of GDPs doing at least some implant procedures, regardless of whether these procedures are surgical or prosthetic, as reported by Gilbert et al.^13^ It can be clearly observed that implant rehabilitation, at least from the general practitioner’s perspective, is much easier for the GDPs themselves to perform without involving a specialist.

Female dentists were more likely to refer patients who required implant surgery. This is in accordance with the findings of Cottrell et al^7^ and Zitzman et al,^23^ who also found that female dentists were more likely than their male counterparts to refer patients who required simple dentoalveolar surgery and dental implants. In the present study, GDP gender differences disappeared regarding the restoration phase of implant therapy, where both genders reported similar patterns of referral. If these gender differences are to be changed, efforts should be made to improve surgical education/training, especially among females, thus increasing the number of surgical providers.^23^

The major reason for patient referral was the complexity of the implant surgery procedure followed by cases in which the patient is medically compromised. The same reasons were found by Coulthard et al^8^ regarding the reasons for referral for oral surgery. This finding is encouraging since we see that GDPs are aware of the complexity of the procedures and in such cases prefer that specialists handle them.

The vast majority of GDPs chose to refer to an oral and maxillofacial surgeon (80%). This agrees with the findings of Cotrell et al,^7^ who reported that GDPs would refer more patients to oral surgeons (50.1%) than to periodontists (31.0%). Different findings were reported by Meraw et al,^16^ who revealed no preference toward periodontists or oral surgeons when implants were placed in partially edentulous jaws. However, a greater percentage of implants were placed by oral surgeons (2/3 of the implants) compared to periodontists (1/3 of the implants). Ghiabi et al^12^ found that periodontists would receive most of the referrals for single and multiple implants and for implants to be placed in the aesthetic zone, while patients needing complex implant-related surgical procedures (i.e., sinus and ridge augmentation or removal of failed implants) were referred more commonly to oral surgeons.

It may be speculated that the reason for more implant surgery referrals to oral and maxillofacial surgeons is due to the referring GDP’s confidence in the oral and maxillofacial surgeon’s ability to control complications when extensive ridge augmentation is necessary. Another reason may be a higher number of practicing oral and maxillofacial surgeons compared to periodontists in our survey setting.

In this study, we did not examine whether the location differences between the GDP and the specialist can influence referral patterns. Linden et al^15^ reported that specialists who practiced further from the sites of GDP services received fewer patient referrals.

The clinic setup did not affect the referral decision. This is in contrast with the findings reported by Field et al^11^ considering the practitioner’s inclination to prefer primary care (private) referral to secondary care (for National Health System (NHS)-funded treatment). It demonstrates that health providers can use both public and private services for implant dentistry.

The limitations of the survey should be emphasised. Althouth 2000 dentists attended the meeting, only 150 initially agreed to participate, and of these, 140 completed the questionnaire. It is difficult to define how accurately these respondents represent the total population of dental practitioners. Unfortunately, specific characteristics of the surveyed dentist population do not exist, making choosing a representative group extremely challenging. Despite these limitations, trends in implant dentistry referral patterns were evident.

It is suggested that formal and informal education/training in implant treatment will improve dental care and increase the total number of dentists practicing both implant surgery and implant prosthetics.

## Conclusions

Most implant surgery is still performed by specialists. Further education/training, especially for female dentists, might increase the rate of implant surgery performed by GDPs. Complex cases will remain for the specialists and can be done in private or public clinics.

## Appendix A Questionnaire

The questionnaire is part of a final student thesis. The purpose of the questionnaire is to characterise the clinical cases referred to specialists by general dental practitioners for implant dentistry. You can choose more than one answer. Thank you for your cooperation.

**Personal Data:**

- Age:
- Gender: Female ☐ Male ☐
- University:
- Years of expertise:
- Postdoctoral studies:

**1. As a general dental practitioner, do you perform implant rehabilitation?**

- A – Yes
- B – No

**2. As a general dental practitioner, do you perform implant surgery?**

- *A – Yes*
- *B – No*

**3. How many times per month do you refer to specialists for implant surgery?**

- A – 0 to 5
- B – 6 to 10
- C – 11 to 15
- D – 16 to 20

**4. Which parameters influence your decision to perform the implant surgery yourself or to refer to a specialist?**

- A – The complexity of the surgical procedure
- B – The compromised medical status of the patient
- C – The high professional competence of the specialist
- D – I do not perform implant surgery on a regular basis
- E – Lack of proper equipment for performing the surgery
- F – I have good cooperation with specialists
- G – Possible surgical complications

**5. To which specialist do you prefer to refer your patient?**

- A – Oral and maxillofacial surgeon
- B – Periodontist
- C – Other (specialty field)

**6. Which medical institution is your preference for referral?**

- A – Hospital (department?)
- B – University clinic (department?)
- C – Private specialist clinic (specialty?)
- D – Location does not matter as long as a specialist provides the treatment

## References

[ref1] American Dental Association Survey Center (2007). The 2005-06 Survey of Dental Services Rendered.

[ref2] American Dental Association Dentistry fact sheet: What career options are available in dentistry?. http://www.ada.org/public/education/careers/dentistry_fact.asp.

[ref3] Buser D, Sennerby L, De Bruyn H (2017). Modern implant dentistry based on osseointegration: 50 years of progress, current trends and open questions. Periodontol 2000.

[ref4] Chaushu L, Tal H, Sculean A, Fernández-Tomé B, Chaushu G (2021). Effects of peri-implant infection on serum biochemical analysis. J Periodontol.

[ref5] Chaushu L, Tal H, Sculean A, Fernández-Tomé B, Chaushu G (2020). Peri-implant disease affects systemic complete blood count values-an experimental in vivo study. Clin Oral Investig.

[ref6] Chaushu L, Weinreb M, Beitlitum I, Moses O, Nemcovsky CE (2015). Evaluation of a topical herbal patch for soft tissue wound healing: an animal study. J Clin Periodontol.

[ref7] Cottrell DA, Reebye UN, Blyer SM, Hunter MJ, Mehta N (2007). Referral patterns of general dental practitioners for oral surgical procedures. J Oral Maxillofac Surg.

[ref8] Coulthard P, Kazakou I, Koron R, Worthington HV (2000). Referral patterns and the referral system for oral surgery care. Part 1: General dental practitioner referral patterns. Br Dent J.

[ref9] Curtis DA, Lin GH, Rajendran Y, Gessese T, Suryadevara J, Kapila YL (2021). Treatment planning considerations in the older adult with periodontal disease. Periodontol 2000.

[ref10] Duong HY, Roccuzzo A, Stähli A, Salvi GE, Lang NP, Sculean A (2022). Oral health-related quality of life of patients rehabilitated with fixed and removable implant-supported dental prostheses. Periodontol 2000.

[ref11] Field JC, Rousseau N, Thomason JM, Exley C, Finch T, Steele JG, Ellis JS (2009). Facilitation of implant provision in primary care. Br Dent J.

[ref12] Ghiabi E, Matthews DC (2012). Periodontal practice and referral profile of general dentists in Nova Scotia, Canada. J Can Dent Assoc.

[ref13] Gilbert GH, Gordan VV, Korelitz JJ, Fellows JL, Meyerowitz C, Oates TW (2015). Provision of specific dental procedures by general dentists in the National Dental Practice-Based Research Network: questionnaire findings. BMC Oral Health.

[ref14] Kolerman R, Qahaz N, Barnea E, Mijiritsky E, Chaushu L, Tal H, Nissan J (2020). Allograft and collagen membrane augmentation procedures preserve the bone level around implants after immediate placement and restoration. Int J Environ Res Public Health.

[ref15] Linden GJ, Stevenson M, Burke FJ (1999). Variation in periodontal referral in 2 regions in the UK. J Clin Periodontol.

[ref16] Meraw SJ, Eckert SE, Yacyshyn CE, Wollan PC (1999). Analysis of surgical referral patterns for endosseous dental implants. Int J Oral Maxillofac Implants.

[ref17] Nissan J, Snir D, Rosner O, Kolerman R, Chaushu L, Chaushu G (2016). Reliability of retrievable cemented implant-supported prostheses. J Prosthet Dent.

[ref18] Nissan J, Zenziper E, Rosner O, Kolerman R, Chaushu L, Chaushu G (2015). The effect of mucosal cuff shrinkage around dental implants during healing abutment replacement. J Oral Rehabil.

[ref19] Renouard F, Renouard E, Rendón A, Pinsky HM (2023; 2023 May 15). Increasing the margin of patient safety for periodontal and implant treatments: The role of human factors. Periodontol 2000.

[ref20] Tomasi C, Derks J (2022). Etiology, occurrence, and consequences of implant loss. Periodontol 2000.

[ref21] Tsaousoglou P, Chatzopoulos GS, Tsalikis L, Lazaridou T, Mikrogeorgis G, Vouros I (2023;17). Prevalence and risk indicators of peri-implantitis: a university based cross-sectional study. Quintessence Int.

[ref22] US Department of Labor Occupational outlook handbook, physicians and surgeons. http://www.bls.gov/oco/ocos074.htm/.

[ref23] Zitzmann NU, Zemp E, Weiger R, Lang NP, Walter C (2011). Does a clinician’s sex influence treatment decisions?. Int J Prosthodont.

